# Recovery of the Acute Hypoxic Ventilatory Response after Reversal of a Minimal Neuromuscular Block: A Randomized Controlled Trial in Healthy, Nonobese Volunteers

**DOI:** 10.1097/ALN.0000000000005650

**Published:** 2025-07-11

**Authors:** Merel A. J. Snoek, Maarten A. van Lemmen, Rutger van der Schrier, Monique van Velzen, Albert Dahan, Martijn Boon

**Affiliations:** 1 Department of Anesthesiology, Leiden University Medical Center, Leiden, The Netherlands.; 2 Department of Anesthesiology, Leiden University Medical Center, Leiden, The Netherlands.; 3 Department of Anesthesiology, Leiden University Medical Center, Leiden, The Netherlands.; 4 Department of Anesthesiology, Leiden University Medical Center, Leiden, The Netherlands.; 5 MediD Consultancy Group, Amsterdam, The Netherlands; Centre for Human Drug Research, Leiden, The Netherlands; Outcomes Research Consortium, Houston, Texas.; 6 Department of Anesthesiology, Leiden University Medical Center, Leiden, The Netherlands.

## Abstract

**Background:**

Neuromuscular blocking agents inhibit the peripheral chemoreflex. This study examined the effect of 2 and 4 mg/kg sugammadex compared to spontaneous recovery of neuromuscular block on the recovery of the acute hypoxic ventilatory response (AHVR).

**Methods:**

This was a two-experiment, randomized, controlled trial in healthy volunteers. Participants received a continuous infusion of rocuronium, to achieve stable symptoms of neuromuscular block in the head and neck region (symptomatic neuromuscular block). Thereafter, neuromuscular block was allowed to recover spontaneously in the first experiment, while in experiment 2, volunteers were randomized to receive 2 mg/kg or 4 mg/kg sugammadex for reversal. The depth of neuromuscular block was assessed with electromyography at the adductor pollicis muscle. AHVR was measured at baseline, during stable neuromuscular block, and at 0, 20, and 40 min after recovery.

**Results:**

A total of 37 volunteers were enrolled; data from 27 volunteers were eligible for analysis. AHVR was reduced by 32% (mean difference *vs.* baseline, –0.22 l · %^−1^ · min^−1^; 95% CI, –0.32 to –0.12) during symptomatic neuromuscular block (mean train-of-four ratio, 0.42 ± 0.22;). At the disappearance of all symptoms, AHVR remained on average depressed by 23% (mean difference, –0.16 l · %^−1^ · min^−1^; 95% CI, –0.28 to –0.04). In 57% of volunteers after spontaneous recovery *versus* 28% after sugammadex reversal, AHVR did not return to baseline values during the measurement period. In addition, the magnitude of residual AHVR depression was greater after spontaneous recovery compared to reversal with sugammadex. However, on average AHVR was not significantly different from baseline at 20 and 40 min after recovery in any group.

**Conclusions:**

The AHVR after reversal of a minimal neuromuscular block with sugammadex did not significantly differ with spontaneous recovery of neuromuscular block. However, fewer patients had residual depression of AHVR when sugammadex was used. In all groups, a considerable proportion of patients had residual depression of the AHVR 40 min after recovery.

## Abstract 2

The hypothesis that 4 mg/kg sugammadex would improve recovery of the peripheral chemoreflex was tested by comparing the effects of 2 and 4 mg/kg sugammadex on the recovery of the acute hypoxic ventilatory response (AHVR) after minimal neuromuscular block with that of spontaneous recovery in a randomized controlled trial in 26 healthy volunteers. Recovery of AHVR lagged behind both the subjective and measured recovery from neuromuscular block. Reversal of neuromuscular block with neither 2 nor 4 mg/kg sugammadex affected recovery of the AHVR, although fewer volunteers experienced prolonged AHVR.

## Editor’s Perspective

**What We Already Know about This Topic**

- Neuromuscular blocking drugs inhibit the peripheral chemoreflex, a hyperventilatory reflex in response to hypoxia, by blocking cholinergic transmission at the carotid bodies
- Neither neostigmine nor 2 mg/kg sugammadex administered for reversal of a minimal, rocuronium-induced neuromuscular block improved the recovery of this reflex

**What This Article Tells Us That Is New**

- The hypothesis that 4 mg/kg sugammadex would improve recovery of the peripheral chemoreflex was tested by comparing the effects of 2 and 4 mg/kg sugammadex on the recovery of the acute hypoxic ventilatory response (AHVR) after minimal neuromuscular block with that of spontaneous recovery in a randomized controlled trial in 26 healthy volunteers
- Recovery of AHVR lagged behind both the subjective and measured recovery from neuromuscular block
- Reversal of neuromuscular block with neither 2 nor 4 mg/kg sugammadex affected recovery of the AHVR, although fewer volunteers experienced prolonged AHVR

Neuromuscular blocking agents are routinely used during general anesthesia to facilitate endotracheal intubation and to optimize surgical working conditions.^1^ However, neuromuscular blocking agents are associated with postoperative respiratory adverse events.^2,3^ Even low levels of residual neuromuscular blocking agents substantially compromise ventilatory control. By blocking neuronal nicotinic acetylcholine receptors at the carotid bodies, neuromuscular blocking agents inhibit the peripheral chemoreflex, which is a life-saving hyperventilatory reflex in response to hypoxia.^4–9^ Intuitively, full restoration of the peripheral chemoreflex is warranted to minimize the chance of hypoxic respiratory events in the postanesthesia care unit. However, in a previous study by our group,^10^ neither neostigmine nor sugammadex (2 mg/kg) significantly improved the recovery of this reflex for reversal of a minimal, rocuronium-induced neuromuscular block.^10,11^ In addition, that study also found that regardless of the reversal strategy, peripheral chemosensitivity remained impaired in greater than 60% of volunteers, at the moment of full recovery of neuromuscular block (*i.e*., train-of-four [TOF] ratio, 1.0). It was hypothesized that a higher sugammadex dose might improve the recovery of the peripheral chemoreflex. In addition, the time relationship of acute hypoxic ventilatory response (AHVR) recovery after recovery of neuromuscular block needed further study. In the current study, the effect of 2 and 4 mg/kg sugammadex on the recovery of the AHVR after minimal neuromuscular block was compared to spontaneous recovery and evaluated up to 40 min after recovery of neuromuscular block.

## Materials and Methods

### Ethics

This single-center, single-blind, two-experiment randomized controlled trial was performed at the Anesthesia and Pain Research Unit of the Department of Anesthesiology at Leiden University Medical Center (The Netherlands) from January 2022 until July 2023. The protocol was approved by the medical ethics committee of the Leiden University Medical Center, METC (Dutch Medical Ethical committee) Leiden-Den Haag-Delft (under identifier P21.083; principal investigator: Martijn Boon), and the Central Committee on Research Involving Human Subjects (competent authority) in The Hague, The Netherlands (identifier NL 78678.058.21). The study protocol and a subsequent substantial amendment were registered in the trial register of the Dutch trial registry, currently available in the World Health Organization International Clinical Trial Registry Platform (https://trialsearch.who.int) under identifier EUCTR2020-000057-27-NL on October 27, 2021, and ClinicalTrials.gov under identifier NCT05149872 (principal investigator: Martijn Boon). The study was conducted following current Good Clinical Practice Guidelines and adhered to the principles of the Declaration of Helsinki.

### Volunteers

Healthy volunteers aged 18 yr or older were eligible for this study. Exclusion criteria were body mass index above 30 kg/m^2^, American Society of Anesthesiologists physical status classification III or higher, gastroesophageal regurgitation, known or suspected neuromuscular disorders impairing neuromuscular function, allergies to neuromuscular blocking agents, anesthetics or opioids, a (self or family) history of malignant hyperthermia or any other muscle disease, and any neurologic or psychiatric illness, including a history of anxiety. Volunteers were not allowed to eat for at least 6 h or drink for at least 2 h before the start of the experiment. All participants gave their written informed consent after receiving verbal and written information about the study and were financially compensated for participating. The experiments were conducted in a controlled laboratory setting. All the volunteers were monitored using a five-lead electrocardiogram, a blood pressure cuff on the upper arm, and pulse oximetry.

### Study Design

The initial study protocol consisted of two visits to our clinic, with two experiments being planned on each visit day. This protocol was substantially amended after the first five volunteers had been enrolled. Volunteers reported that in the initial protocol, the experiment day was long and physically demanding, which resulted in a high rate of premature stops. We also observed no meaningful effect on the hypercapnic ventilatory response in the first volunteers during partial neuromuscular block (data not shown), which is in line with previous findings by Broens *et al.*^10^ Therefore, the protocol was amended to improve the tolerability for the volunteers as follows: (1) the number of experiments was reduced from four to two, allowing participants to complete the study within one day; (2) hypercapnic ventilatory response measurements were removed from the protocol; and (3) the approach to rocuronium titration was altered: rather than targeting a specific TOF ratio, the titration was now aimed at specific neuromuscular block symptoms. The rationale for this came from the observation that TOF ratios varied significantly among volunteers displaying similar symptoms of muscle weakness, which has been observed by others before.^12^ In addition, we observed that symptoms of muscle weakness significantly preceded the decrease of the TOF ratio measured at the thumb. We reasoned that titrating rocuronium based on symptomatology, rather than TOF ratio, would induce a similar level of partial neuromuscular block in all volunteers. This approach improved the tolerability and feasibility of the experiments, as overshoots in muscle weakness did not occur in the amended protocol, and the dropout rate was significantly reduced.

### Study Procedures

In all experiments, a continuous infusion of diluted rocuronium (1 mg/ml) was used to induce neuromuscular block. We initiated the infusion rate of 0.8 mg · kg^−1^ · h^−1^ and reduced the dose to 0.4 mg · kg^−1^ · h^−1^ after 3 min. Subsequently, the rocuronium infusion was titrated to obtain moderate symptoms of muscle weakness (*i.e.*, symptomatic neuromuscular block) in accordance with the amended protocol. The targeted symptoms of neuromuscular block included diplopia, inability to raise the leg for 10 s, or inability to retain a tongue depressor stick between the incisor teeth without having difficulty swallowing. To ensure consistency in evaluation, these tests were performed by the same researcher. Neuromuscular function was monitored using electromyography (GE-EMG; GE Healthcare, USA) at the adductor pollicis muscle, similar to our previous protocol.^10^ TOF measurements were recorded at 1-min intervals using a stimulation current of 30 mA. Before initiating rocuronium infusion, we confirmed the reliability of the electromyography signal by obtaining three consecutive TOF ratio measurements of 100% in each participant.^13^

In the first experiment, the neuromuscular block was allowed to recover spontaneously. In the second experiment, either 2 or 4 mg/kg sugammadex was administered for reversal of symptomatic neuromuscular block. The dosing of sugammadex was determined by randomization, which was performed before the experiments, by a computer-generated randomization list. Only volunteers were blinded to randomization to prevent reporting bias, as knowledge of dose assignment could potentially influence subjective symptom resolution assessments. To minimize carryover effects between the experiments, a 30-min pause was used.

**Fig. 1.**
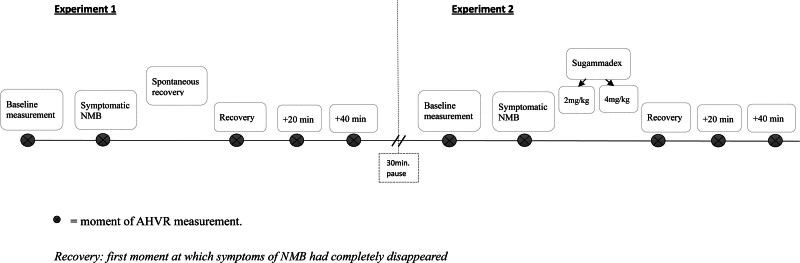
Interventions and measurements of acute hypoxic ventilatory response (AHVR). The moment of AHVR measurement is indicated by a *circled ×*. NMB, neuromuscular block.

In both experiments, the AHVR was measured at baseline (*i.e.*, before the start of rocuronium infusion), during stable symptomatic neuromuscular block, at the first moment of complete recovery of all symptoms, and at 20 and 40 min after recovery (see fig. 1). The methodology of the ventilatory measurements was similar to the methodology of our previous study.^10^ Briefly, we obtained the isocapnic hypoxic ventilatory response by rapidly reducing the fractional inspired oxygen tension to 0.08, aiming to achieve an oxygen saturation level of 80 ± 2%, as measured by pulse oximetry. The hypoxic test continued until the hyperventilatory response plateaued for at least 2 min. Throughout the experiment, the end-tidal CO_2_ was maintained at 2.3 mmHg above resting values. Hypoxic ventilatory sensitivity was calculated by linear regression of the arterial oxygen saturation – minute ventilation (V̇_E_) data using 2 min of stable ventilation in normoxia and hypoxia.

### Statistical Analysis

The primary endpoint was for the study was the time at which the AHVR had returned to the baseline value. Secondary endpoints included the sugammadex dose–response relationship for AHVR recovery and the relationship of AHVR to clinical symptoms and TOF ratio. We hypothesized that sugammadex reversal would result in faster recovery of AHVR compared to spontaneous recovery of neuromuscular block and that this effect would be dose dependent. As there was no previous data to base our sample on, we defined a 50% faster recovery of AHVR with sugammadex reversal compared to spontaneous recovery to be clinically relevant. However, it must be appreciated that this is an exploratory study. With α set at 0.05 and β at 0.2, the sample size calculation resulted in 33 participants (two-sided paired *t* test). Although the protocol was amended as explained earlier, we opted to maintain the planned sample size.

We analyzed AHVR using a two-level linear mixed-effects model to account for repeated measures within subjects. Fixed effects included baseline AHVR, a categorical experiment factor (spontaneous recovery, 2 mg/kg sugammadex, or 4 mg/kg sugammadex), a categorical timepoint factor (baseline, symptomatic neuromuscular block, recovery of symptomatic neuromuscular block, recovery + 20 min, or recovery + 40 min), and their interaction. We fit the model by restricted maximum likelihood in *R* (version 4.3.0) with the lme4 package (version 1.1-31),^14^ specifying a random intercept for each subject. Statistical inference for fixed effects was obtained *via* Satterthwaite’s approximation using the emmeans package (version 1.8.4).^14^ β estimates and variance estimates of the model are given in Supplemental Digital Content 1 (https://links.lww.com/ALN/E122).

After fitting the model, we calculated the estimated marginal means (EMMs) for each measurement moment. These EMMs provide a concise summary of the models’ estimates at each of these levels, considering the random effects structure inherent in our data.

To assess the statistical significance of the variations in AHVR between the baseline and the other measurements, we performed pairwise contrasts of these EMMs. This analysis facilitated a detailed examination of the changes in AHVR from baseline at each subsequent stage. To mitigate the risk of type I error due to multiple comparisons, a Tukey adjustment was applied to the *P* values of these differences.

## Results

**Fig. 2.**
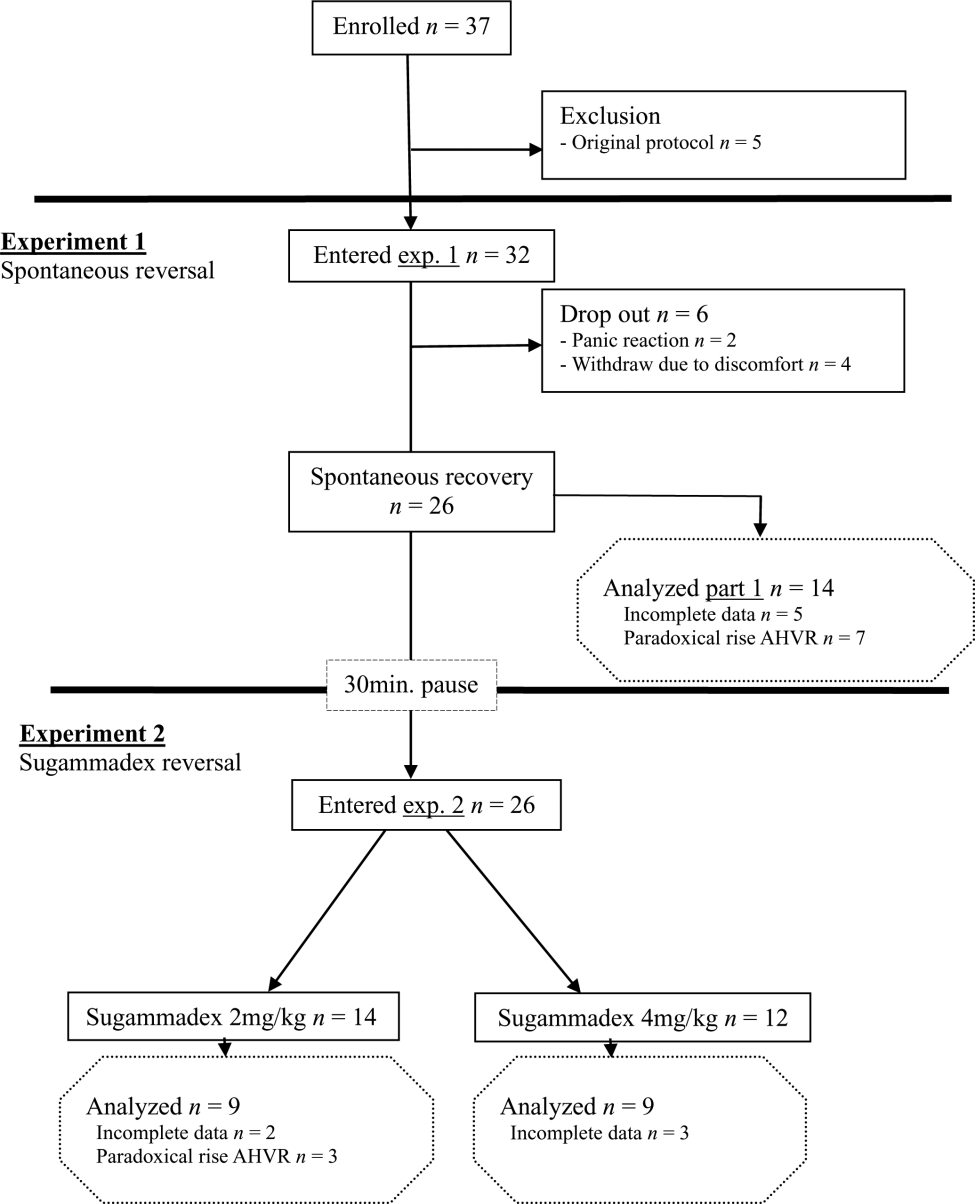
Flowchart. AHVR, acute hypoxic ventilatory response; exp., experiment.

A total of 37 volunteers were enrolled in the study, of whom 32 were randomized and received intervention according to the amended protocol. Two volunteers with a panic reaction were given sugammadex prematurely; these two volunteers were replaced. Additionally, four volunteers withdrew from the study due to discomfort with the first neuromuscular block dosing (fig. 2). Data from 10 experiments were excluded from analysis because of a paradoxical increase of AHVR during symptomatic neuromuscular block compared to baseline, due to discomfort or anxiety. Data from 14 and 18 volunteers who completed the entire experiment were used for the final analysis of experiments 1 and 2, respectively (fig. 2). Participant characteristics are shown in table 1.

**Table 1.** Participant Characteristics

Characteristic	Spontaneous Recovery (n = 14)	2 mg/kg Sugammadex (n = 9)	4 mg/kg Sugammadex (n = 9)	All Data (n = 21)
Sex				
Male	5 (35.7%)	4 (44.4%)	5 (55.6%)	10 (47.6%)
Female	9 (64.3%)	5 (55.6%)	4 (44.4%)	11 (52.4%)
Age, yr				
Mean ± SD	36.4 ± 23.8	48.4 ± 25.2	39.3 ± 25.8	40.7 ± 24.6
Median [minimum, maximum]	23.0 [20.0, 76.0]	67.0 [21.0, 73.0]	23.0 [21.0, 76.0]	23.0 [20.0, 76.0]
Body mass index, kg/m^2^				
Mean ± SD	22.0 ± 2.39	21.6 ± 2.44	22.9 ± 2.21	21.9 ± 2.49
Median [minimum, maximum]	21.9 [17.5, 26.3]	21.4 [17.1, 26.1]	23.7 [19.6, 26.3]	21.8 [17.1, 26.3]

### Experiments 1 and 2

**Fig. 3.**
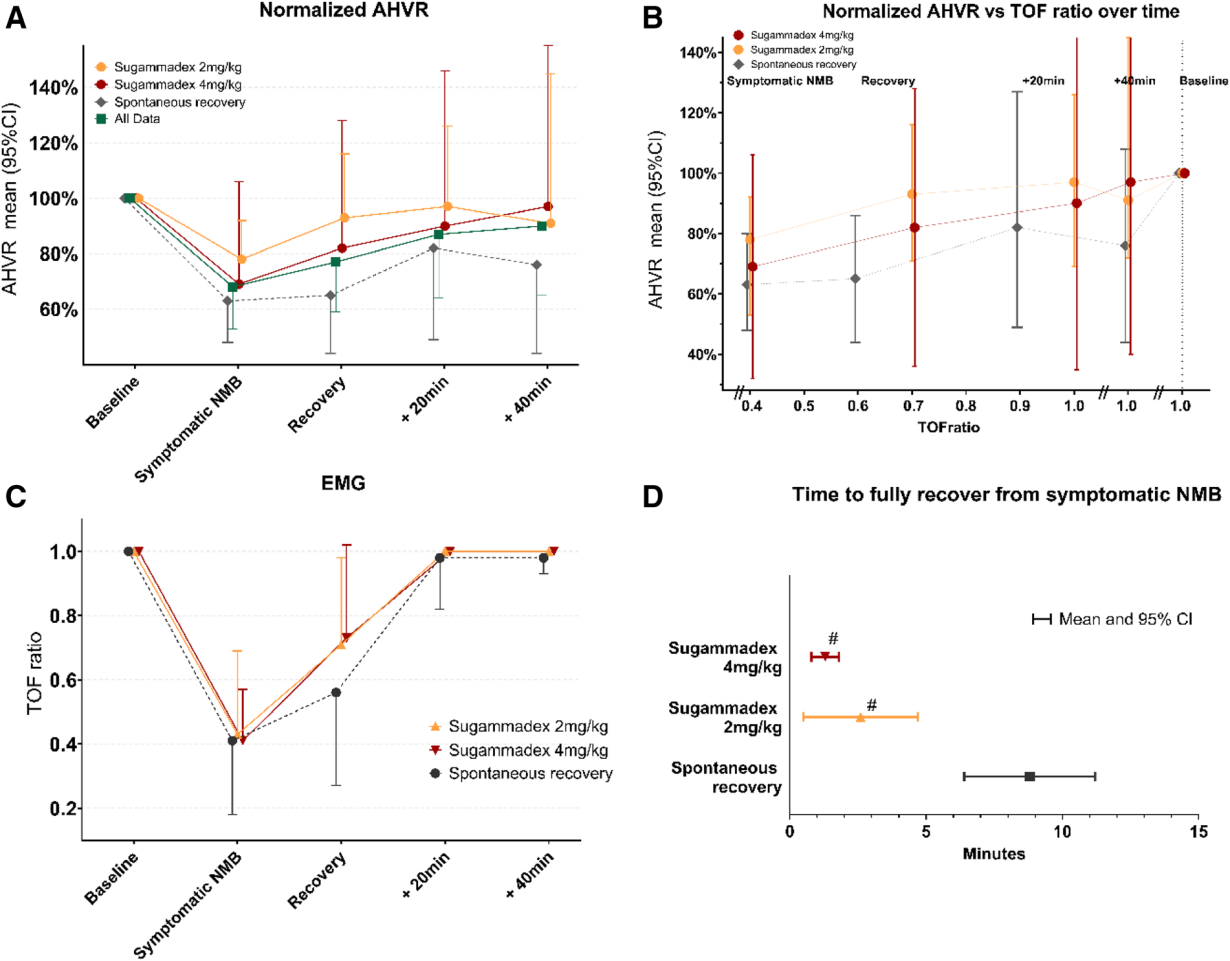
Acute hypoxic ventilatory response (AHVR) and train-of-four (TOF) ratio. Recovery is defined as the first moment at which symptoms of neuromuscular block (NMB) completely disappeared (mean and 95% CI). (*A*) AHVR differences of all groups combined (mean and 95% CI). (*B*) AHVR differences per group (mean and 95% CI). (*C*) Electromyography (EMG) presenting TOF ratio (mean and SD). (*D*) Time to recovery from neuromuscular block symptoms in minutes (mean and 95% CI). #*P* < 0.001 *versus* spontaneous recovery.

Data from both experiments combined showed that the AHVR was reduced by 32% during symptomatic neuromuscular block (mean TOF ratio, 0.42 ± 0.22) *versus* baseline (mean difference [MD] *vs.* baseline, –0.22 l · %^−1^ · min^−1^; 95% CI, –0.32 to –0.12; *P* = 0.0001). At the recovery of symptomatic neuromuscular block, the TOF ratio was 0.66 ± 0.29, and AHVR remained depressed by 23% *versus* baseline (MD, –0.16 l · %^−1^ · min^−1^; 95% CI, –0.28 to –0.04; *P* = 0.010). AHVR remained on average 24% below baseline, even after 20 and 40 min after recovery of symptomatic neuromuscular block, while the TOF ratio had recovered up to 0.96 ± 0.13 after 20 min and 0.99 ± 0.04 after 40 min (table 2; fig. 3A).

**Table 2.** AHVR and TOF Ratio

Data	Baseline	Symptomatic Neuromuscular Block	Recovery	+20 min	+40 min
All data (n = 32)					
TOF ratio (range)	1.0	0.42 ± 0.22 (0.13–0.97)	0.65 ± 0.29 (0.21–1.0)	0.96 ± 0.12 (0.49–1.0)	0.99 ± 0.04 (0.79–1.0)
AHVR, L · %^−1^ · min^−1^	0.69 ± 0.45	0.47 ± 0.29	0.53 ± 0.36	0.61 ± 0.46	0.63 ± 0.49
Mean difference (95% CI)		−0.22 (−0.32, −0.12)	−0.16 (−0.28, −0.04)	−0.09 (−0.25, 0.08)	−0.07 (−0.24, 0.1)
*P* value *vs*. BL		0.0001	0.010	0.396	0.642
Percentage of AHVR *vs*. BL		68	77	87	90
Spontaneous recovery (n = 14)					
TOF ratio (range)	1.0	0.41 ± 0.23 (0.15–0.97)	0.56 ± 0.29 (0.21–0.98)	0.90 ± 0.17 (0.49–1.0)	0.98 ± 0.05 (0.79–1.0)
AHVR, l · %^−1^ · min^−1^	0.71 ± 0.42	0.45 ± 0.22	0.46 ± 0.29	0.58 ± 0.44	0.54 ± 0.44
Mean difference (95% CI)		−0.26 (−0.37, −0.14)	−0.25 (−0.4, −0.1)	−0.13 (−0.36, 0.09)	−0.17 (−0.4, 0.06)
*P* value *vs*. BL		0.048	0.061	0.571	0.319
Percentage of AHVR *vs*. BL		63	65	82	76
2 mg/kg sugammadex (n = 9)					
TOF ratio (range)	1.0	0.45 ± 0.27 (0.13–0.83)	0.73 ± 0.27 (0.32–1.0)	1.0 (–)	1.0 (–)
AHVR, l · %^−1^ · min^−1^	0.58 ± 0.26	0.44 ± 0.21	0.54 ± 0.19	0.56 ± 0.25	0.63 ± 0.32
Mean difference (95% CI)		−0.13 (−0.27, 0.01)	−0.04 (−0.17, 0.09)	−0.02 (−0.18, 0.15)	0.05 (−0.16, 0.26)
*P* value *vs*. BL		0.229	0.967	0.999	0.915
Percentage of AHVR *vs*. BL		78	93	97	91
4 mg/kg sugammadex (n = 9)					
TOF ratio (range)	1.0	0.42 ± 0.16 (0.20–1.0)	0.72 ± 0.29 (0.33–1.0)	1.0 (–)	1.0 (–)
AHVR, l · %^−1^ · min^−1^	0.78 ± 0.63	0.53 ± 0.44	0.63 ± 0.55	0.70 ± 0.66	0.76 ± 0.68
Mean difference (95% CI)		−0.24 (−0.53, 0.05)	−0.14 (−0.5, 0.22)	−0.08 (−0.51, 0.36)	−0.02 (−0.47, 0.43)
*P* value *vs*. BL		0.026	0.363	0.860	0.999
Percentage of AHVR *vs*. BL		69	82	90	97

The values are shown as the ratio means ± SD for TOF and the means ± SD for AHVR.

AHVR, acute hypoxic ventilatory response; BL, baseline; TOF, train of four.

#### Experiment 1: Spontaneous Recovery

In experiment 1, the mean ± SD TOF ratio during stable symptomatic neuromuscular block was 0.41 ± 0.23, however, with large interindividual variation (range, 0.15 to 0.97), and the AHVR was decreased by 37% at this point (MD, −0.26 l · %^−1^ · min^−1^; 95% CI, −0.37 to −0.14; *P* = 0.048). It took on average 8.8 min (range, 3 to 20 min) for all symptoms of neuromuscular block to disappear; however, the mean TOF ratio was 0.56 ± 0.29 at this point, and the mean difference of AHVR *versus* baseline was −0.25 (95% CI, −0.4 to −0.1; *P* = 0.061), reflecting a persistent 35% reduction in AHVR compared to baseline at this point (table 2; fig. 3B). At 20 and 40 min after recovery of symptomatic neuromuscular block, mean AHVR had on average fully recovered and did not significantly differ from baseline values. TOF ratios at these times both were 0.98 ± 0.05. However, in eight volunteers, AHVR was still 30% below baseline at 40 min after recovery (table 3).

**Table 3.** Number of Volunteers and Magnitude of Residual AHVR Depression in Volunteers without Full Recovery of AHVR

	Number of Measurements AHVR below Baseline, n (%)	AHVR, % of Baseline
All data (*n* = 32)	13 (40.6)	76.0 ± 20.0
Spontaneous recovery (*n* = 14)	8 (57.1)	69.7 ± 24.3
All reversals by sugammadex (*n* = 18)	5 (27.8)	84.7 ± 7.2
2 mg/kg sugammadex (*n* = 9)	2 (22.2)	82.2 ± 9.7
4 mg/kg sugammadex (*n* = 9)	3 (33.3)	86.4 ± 0.8

AHVR, acute hypoxic ventilatory response.

#### Experiment 2: Sugammadex Reversal (2 or 4 mg/kg)

In experiment 2, the mean TOF ratio during symptomatic neuromuscular block was 0.44 ± 0.21; there was no significant difference in TOF ratio between the 2 and 4 mg/kg sugammadex groups (fig. 3C). Sugammadex significantly accelerated the time for the symptoms of neuromuscular block to disappear: 2.6 ± 3.3 (range, 0 to 11) min for the 2-mg/kg dose and 1.3 ± 0.7 (range, 0 to 2) min for the 4-mg/kg dose (fig. 3D). The mean ± SD TOF ratios at the disappearance of symptomatic neuromuscular block were 0.73 ± 0.27 and 0.72 ± 0.29, respectively. Notably, 40 min after the disappearance of symptomatic neuromuscular block, 22.2% of volunteers who received sugammadex 2 mg/kg, and 33.3% of volunteers who had received 4 mg/kg still had an AHVR that was 18 and 14% below baseline, respectively (table 3). TOF ratios at this time point were 1.0 in all volunteers.

## Discussion

Neuromuscular blocking agents are routinely administered during general anesthesia; however, lingering effects precipitate respiratory complications in the postanesthesia care unit and on the ward. Attenuation of the peripheral chemoreflex by these agents is an important factor in the pathophysiology of postoperative hypoxic adverse events. Eriksson *et al.*^4–7,15^ were the first to demonstrate that nondepolarizing neuromuscular blocking agents suppress the acute ventilatory response to hypoxia (AHVR) by blocking nicotinic acetylcholine receptors in the carotid bodies. Because cholinergic neurotransmission is critical for signal transduction between the oxygen-sensing type 1 glomus cell and the afferent nerve, inhibition of these receptors markedly reduces peripheral chemotransmission.^16^ In a previous study by our group, a minimal rocuronium-induced neuromuscular block resulted in a 42% reduction in AHVR. Importantly, AHVR remained significantly depressed in a subset of patients even at the point of full recovery from neuromuscular blockade.^10^ In the current study, we similarly observed that AHVR did not return to baseline in 41% of volunteers at 40 min after recovery of symptomatic neuromuscular block (TOF ratio, 1.0). While reversal with sugammadex (2 or 4 mg/kg) did not significantly alter the magnitude of AHVR relative to no reversal, both the previous and current findings indicate that AHVR recovers faster with fewer patients showing incomplete recovery of AHVR when sugammadex is used.

The results of the current and our previous study suggest that the recovery of the peripheral chemoreflex after rocuronium-induced neuromuscular block is poorly reflected by recovery of neuromuscular block measured at the adductor pollicis muscle. Differences in local blood flow, local acetylcholine receptor density, and subtype expression play a role. In contrast to the neuromuscular junction, only the neuronal subtype acetylcholine receptors (nAChR) subtype are expressed in the carotid body, and the variety of subtypes is larger. The activation–inactivation kinetics, as well as the affinity of rocuronium for the various nAChR subtypes, differs substantially, which has important consequences.^17^ For instance, at the neuromuscular junction, a large safety margin exists, meaning that no effect of the neuromuscular blocking agent is observable until more than 75% of the nAChRs are blocked.^18^ In contrast, a linear, dose-dependent depression of carotid sinus nerve activity was seen with neuromuscular blocking agents atracurium and vecuronium, suggesting that no margin of safety exists at the carotid body.^6^ This renders the carotid body more susceptible to low (residual) concentrations of neuromuscular blocking agents, potentially explaining the lagging recovery of the peripheral chemoreflex in comparison to the adductor pollicis muscle. Finally, apart from acetylcholine, type I cells of the carotid body release a variety of other neurotransmitters in response to hypoxemia, including but not limited to dopamine, norepinephrine, serotonin, purines, and neuropeptides.^8,19^ Although a definitive consensus on the exact mechanisms of neurotransmission within the carotid body is lacking, it is clear that cholinergic neurotransmission is only one component of chemotransmission.

Notably, neuromuscular blocking agents are not the only pharmacologic agents that are used in routine anesthesia practice that impair the peripheral chemoreflex. For instance, propofol has shown to interact both with nAChR and with voltage-gated calcium channels at the type 1 cell, while inhalational anesthetics directly interfere with background potassium channels at the type 1 cell, all contributing to inhibition of type 1 cell depolarization in response to hypoxemia.^20^ The combined effects of neuromuscular blocking agents and volatile or intravenous anesthetics on the AHVR in clinical practice have not yet been studied. However, they are hypothesized to be additive or synergistic, warranting further investigation.

In addition to the effects observed on the AHVR, other important observations of neuromuscular block and symptomatology were observed in this study. First, the variability in the TOF ratio between volunteers experiencing the same degree of muscle weakness symptoms was remarkable. In the current protocol, we aimed to achieve a similar degree of neuromuscular block by titrating the neuromuscular block based on symptoms of muscle weakness, instead of a fixed TOF ratio; volunteers needed to fail a 10-s leg raise test or the fail retaining a tongue depressor between the incisor teeth. The TOF ratio for this specific symptomatology varied between 0.13 and 0.97. Importantly, the ability to swallow normally was not affected at these levels of neuromuscular block.^21^ These observations bear important lessons for clinical practice as they indicate that any TOF ratio less than 0.9 could accompany profound muscle weakness in one person, while not causing any symptoms of muscle weakness in other patients. Clinicians should therefore always quantitatively monitor neuromuscular block and always ensure that the TOF ratio has recovered to values of 0.9 or greater before tracheal extubation. The second noteworthy observation was the time delay between the onset and offset of symptoms of muscle weakness and the measured TOF ratio at the adductor polices muscle. It was our experience that patients started to experience symptoms of diplopia within minutes after the start of infusion of rocuronium. Diplopia slowly progressed toward more profound muscle weakness in about 30 to 40 min. However, the TOF ratio commonly only started to decrease 30 min after the start of the infusion. *Vice versa*, when the infusion was stopped, most volunteers reported relief of symptoms within minutes, while diplopia could last 10 min or longer in the situation without active reversal. The TOF ratio at resolution of symptoms was on average 0.72 and remained depressed for a significant amount of time despite the clinical resolution of all symptoms. Again, the clinical consequence of these findings is that the absence of symptoms of muscle weakness does not equal a TOF ratio greater than 0.9 measured at the adductor polices muscle nor a full recovery of the peripheral chemoreflex. Finally, our protocol for infusing rocuronium has been shown to be safe for volunteers, with no volunteers requiring an emergency reversal due to respiratory adverse events.

Our study has several limitations. First, we arbitrarily stopped measurements at 40 min after full recovery of neuromuscular block to keep the experiment compact. However, many volunteers still showed incomplete recovery of AHVR at that time. Second, we amended the protocol to guide the rocuronium on the development of clinical symptoms rather than a specific TOF ratio range; our results may not be readily comparable with protocols that did not use this technique. Third, we did not assess the hypercapnic ventilatory response to evaluate the potential contribution of respiratory muscle weakness, as these experiments were poorly tolerated by the volunteers due to their prolonged duration in addition of the hypoxic measurements and the demanding nature. Moreover, initial findings from this study, in conjunction with our previous results, suggest minimal effects on the diaphragm and, consequently, on the effort-driven component of breathing. Fourth, we excluded data from volunteers that showed a paradoxical rise (instead of a decrease) in AHVR during partial neuromuscular block (shown in Supplemental Digital Content 2, https://links.lww.com/ALN/E123). Similar to what has been observed and suggested before, discomfort or anxiety that resulted from pain inflicted by the TOF measurements or the perception of muscle weakness may have influenced ventilation at the moment of measuring AHVR in these volunteers, leading to a biased measurement result.^22,23^ Analysis of the full data set is given in Supplemental Digital Content 3 (https://links.lww.com/ALN/E124) and similarly shows no effect of reversal with sugammadex *versus* no reversal. Fifth, we cannot rule out that a small carryover effect between experiments 1 and 2 may have existed. To account for potential changes in participant status, including factors such as fatigue or other time-dependent variations such as hunger or restlessness, a new baseline measurement was taken at the start of experiment 2. The fact that the two baselines were grossly similar suggests that no meaningful carryover effect of rocuronium on the peripheral chemoreflex existed. Finally, it should be noted that the data came from healthy volunteers who were tested in an experimental setting. It is likely that in the clinical setting, the attenuation of AHVR may be worse, and it may take longer for AHVR to recover. It is known that both intravenous and inhalational hypnotics directly inhibit peripheral chemosensitivity by various mechanisms.^22,24,25^ In addition, opioids centrally inhibit afferent projection of peripheral chemosensitivity in the brainstem. As hypnotics, opioids, and neuromuscular blocking agents are the cornerstone pharmaceutics of general anesthesia; the combined lingering effects of these agents on peripheral chemosensitivity are potentially significantly worse than the effects of any of these agents alone.^20,26^

In conclusion, this study showed that the AHVR is depressed during minimal neuromuscular block and that recovery of this response lags behind both the subjective and measured recovery of neuromuscular block. Reversal with 2 or 4 mg/kg sugammadex did not significantly affect the recovery of the AHVR, although it resulted in fewer patients having depression of the AHVR. The relevance of this finding should be further studied in a clinical setting. Future experiments on muscle relaxation in awake volunteers should consider implementing our methodology.

### Research Support

Supported in part by a research grant from Investigator-Initiated Studies Program of Merck Sharp & Dohme LLC, a subsidiary of Merck & Co., Inc. (Rahway, New Jersey).

### Competing Interests

Dr. Dahan received consultancy fees from Trevena Inc. (Atlanta, Georgia), Zmi Pharma (Carlsbad, California), and Enalare Therapeutics (Princeton, New Jersey) outside of the content of the topic of this work. The other authors declare no competing interests.

### Reproducible Science

Full protocol available at: m.a.j.snoek@lumc.nl. Raw data available at: m.a.j.snoek@lumc.nl.

## Supplemental Digital Content

Supplement 1. Mixed-effects model estimate, https://links.lww.com/ALN/E122

Supplement 2. Excluded experiment per subject ID, https://links.lww.com/ALN/E123

Supplement 3. Analysis of the full data-set, https://links.lww.com/ALN/E124

## Supplementary Material
