# Supplementary material for: Recovery of the Acute Hypoxic Ventilatory Response after Reversal of a Minimal Neuromuscular Block: A Randomized Controlled Trial in Healthy, Nonobese Volunteers
Source: Anesthesiology. 2025 Jul 11;143(4):873–82. doi: 10.1097/ALN.0000000000005650 (PMC12416893; doi:10.1097/ALN.0000000000005650)
Supplement: Supplementary file 1 [file aln-143-873-s001.pdf]

Supplement material 1:

Table Mixed-effects model estimates

Fixed effects	Estimate (β)	Std. Error	t value	95 % CI
(Intercept)	0.18633	0.06008	3.102	[0.0687, 0.3040]
**Spontaneous recovery**				
Baseline	0.71726	0.05432	13.204	[0.6108, 0.8238]
Symptomatic NMB	–0.12848	0.06148	–2.090	[–0.2490, –0.0079]
Recovery	–0.17161	0.06148	–2.791	[–0.2921, –0.0511]
+ 20 min	–0.07732	0.06148	–1.258	[–0.1978, 0.0432]
Recovery + 40 min	–0.10260	0.06148	–1.669	[–0.2239, 0.0187]
**Sugammadex 2mg/kg**				
Baseline	–0.03943	0.07328	–0.538	[–0.1831, 0.1042]
Symptomatic NMB	0.05468	0.10195	0.536	[–0.1452, 0.2545]
Recovery	0.13314	0.10195	1.306	[–0.0667, 0.3330]
+ 20 min	0.05652	0.10195	0.554	[–0.1434, 0.2564]
+ 40 min	0.15726	0.10195	1.542	[–0.0426, 0.3571]
**Sugammadex 4mg/kg**				
Baseline	0.03666	0.08111	0.452	[–0.1223, 0.1957]
Symptomatic NMB	–0.11473	0.11225	–1.022	[–0.3347, 0.1052]
Recovery	0.02929	0.11225	0.261	[–0.1907, 0.2492]

Fixed effects	Estimate (β)	Std. Error	t value	95 % CI
+ 20 min	0.00159	0.11225	0.014	[–0.2184, 0.2215]
+ 40 min	0.08383	0.11225	0.747	[–0.1361, 0.3038]
