# Supplementary material for: Recovery of the Acute Hypoxic Ventilatory Response after Reversal of a Minimal Neuromuscular Block: A Randomized Controlled Trial in Healthy, Nonobese Volunteers
Source: Anesthesiology. 2025 Jul 11;143(4):873–82. doi: 10.1097/ALN.0000000000005650 (PMC12416893; doi:10.1097/ALN.0000000000005650)

Supplemental material 2:

The excluded experiment per subject ID due to a paradoxical rise during symptoms of neuromuscular blockade.

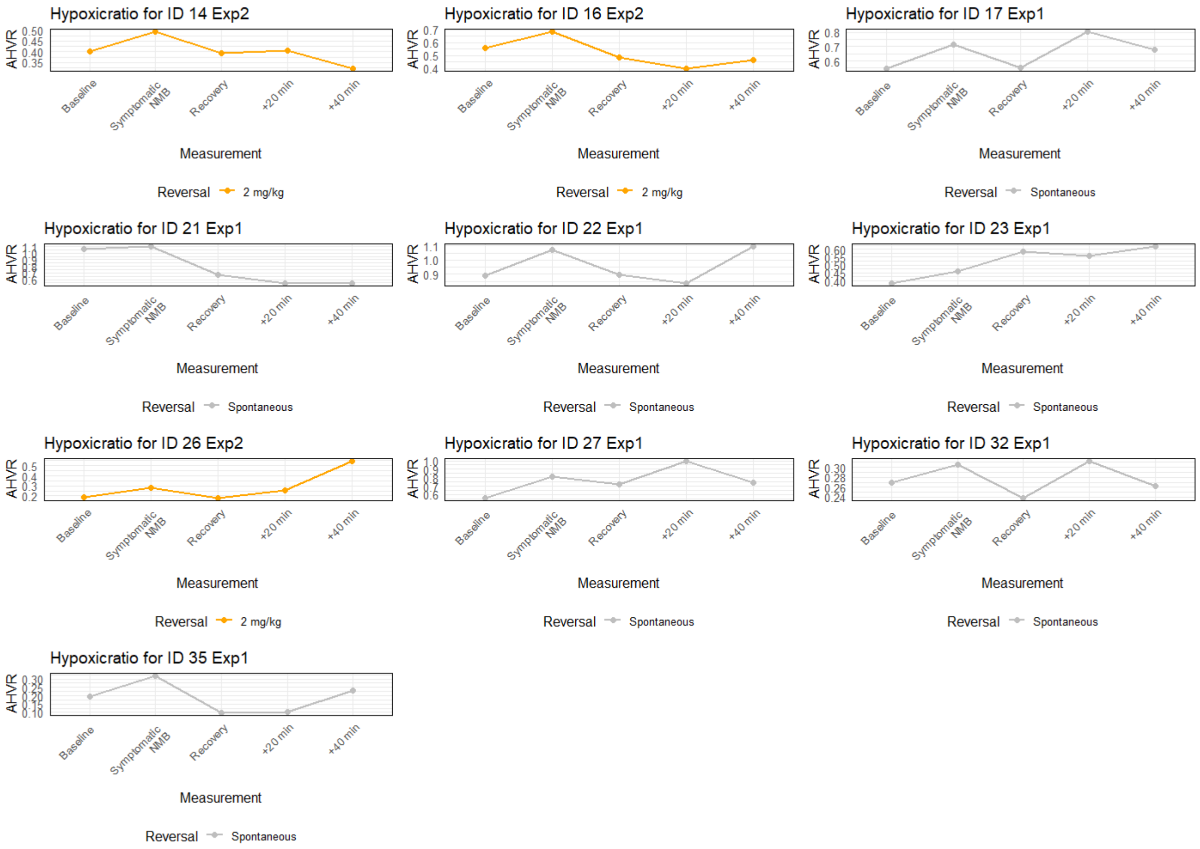

Supplement: Supplementary file 2 [file aln-143-873-s002.pdf]
