# Supplementary material for: Recovery of the Acute Hypoxic Ventilatory Response after Reversal of a Minimal Neuromuscular Block: A Randomized Controlled Trial in Healthy, Nonobese Volunteers
Source: Anesthesiology. 2025 Jul 11;143(4):873–82. doi: 10.1097/ALN.0000000000005650 (PMC12416893; doi:10.1097/ALN.0000000000005650)
Supplement: Supplementary file 3 [file aln-143-873-s003.pdf]

Supplemental material 3:

Analysis of the full data-set, including 10 excluded experiments that were excluded for the final analysis because of a paradoxical increase of AHVR during symptomatic NMB.

	Baseline	Symptomatic NMB	Recovery	+20min	+40min
**All experiments (n = 42)**					
AHVR [L.%$^{-1}$.min^{-1}] *(95% CI)*	0.65 (0.57, 0.73)	0.50 (0.42,0.58)	0.53 (0.46,0.61)	0.59 (0.51, 0.67)	0..63 (0.55, 0.70)
Mean difference *(95% CI,) p-value*		-0.15 (-0.27, -0.03), *p*=0.006 *vs*. BL	-0.12 (-0.23, -0.001), *p*=0.047 *vs*. BL	-0.06 (-0.17, 0.06), *p*=0.61 *vs*. BL	-0.02 (-0.14, 0.09), *p*=0.98 *vs*. BL
**Spontaneous recovery (n= 21)**					
AHVR *(L.%$^{-1}$.min^{-1})*	0.65 (0.56, 0.75)	0.52 (0.43, 0.62)	0.48 (0.38, 0.57)	0.57 (0.48, 0.67)	0.55 (0.45, 0.64)
Mean difference *(95% CI,) p-value*		-0.13 (-0.28, 0.02), *p*=0.14 *vs*. BL	-0.17 (-0.33, -0.02), *p*=0.02 *vs*. BL	-0.08 (-0.23, 0.08), *p*=0.61 *vs*. BL	-0.10 (-0.26, 0.05), *p*=0.33 *vs*. BL
**Sugammadex 2 mg/kg (n=12)**					
AHVR *(L.%$^{-1}$.min^{-1})*	0.61 (0.49, 0.74)	0.0.54 (0.41, 0.66)	0.57 (0.45, 0.70)	0.59 (0.47, 0.71)	0.67 (0.54, 0.79)
Mean difference *(95% CI,) p-value*		-0.07 (-0.28, 0.13), *p*=0.83 *vs*. BL	-0.04 (-0.24, 0.17), *p*=0.98 *vs*. BL	-0.02 (-0.2, 0.18), *p*=1.0 *vs*. BL	0.05 (-0.15, 0.26), *p*=0.94 *vs*. BL
**Sugammadex 4 mg/kg (n= 9)**					
AHVR *(L.%$^{-1}$.min^{-1})*	0.69 (0.55, 0.83)	0.44 (0.30, 0.59)	0.55 (0.40, 0.69)	0.61 (0.47, 0.75)	0.67 (0.53, 0.81)
Mean difference *(95% CI,) p-value*		-0.24 (-0.48, -0.007), *p*=0.02 *vs*. BL	-0.14 (-0.38, 0.09), *p*=0.36 *vs*. BL	-0.08 (-0.31, 0.16), *p*=0.86 *vs*. BL	-0.02 (-0.26, 0.22), *p*=1.0 *vs*. BL

AHVR estimated mean (95% CI), mean difference (95% CI), p-value compared to baseline value.

Adding data from previously excluded experiments led to a uniform shift in the estimated marginal means across all experimental levels—including the level that was originally complete—so that while the absolute values changed, the pairwise contrasts (differences from baseline) remained unchanged.
